# Physiological and Enzymatic Antioxidant Responses of *Solanum tuberosum* Leaves to Arbuscular Mycorrhizal Fungal Inoculation under Water Stress

**DOI:** 10.3390/plants13081153

**Published:** 2024-04-21

**Authors:** Javiera Nahuelcura, Catalina Bravo, Analía Valdebenito, Sheina Rivas, Christian Santander, Felipe González, Pablo Cornejo, Boris Contreras, Antonieta Ruiz

**Affiliations:** 1 Departamento de Ciencias Químicas y Recursos Naturales, Scientific and Technological Bioresource Nucleus BIOREN-UFRO, Universidad de La Frontera, Avenida Francisco Salazar 01145, Temuco 4811230, Chilec.santander01@ufromail.cl (C.S.);; 2 Doctorado en Ciencias de Recursos Naturales, Universidad de La Frontera, Temuco 4811230, Chile; 3 Doctorado en Ciencias Mención Biología Celular y Molecular Aplicada, Universidad de La Frontera, Temuco 4811230, Chile; 4 Escuela de Agronomía, Facultad de Ciencias Agronómicas y de los Alimentos, Pontificia Universidad Católica de Valparaíso, Quillota 2260000, Chile; pablo.cornejo@pucv.cl; 5 Centro Regional de Investigación e Innovación para la Sostenibilidad de la Agricultura y los Territorios Rurales, CERES, La Palma, Quillota 2260000, Chile; 6 Novaseed Ltd., Loteo Pozo de Ripio s/n, Parque Ivian II, Puerto Varas 5550000, Chile

**Keywords:** potato, water stress, antioxidant enzyme, mycorrhiza

## Abstract

*Solanum tuberosum* is one of the most widely cropped plant species worldwide; unfortunately, drought is one of the major constraints on potato productivity because it affects the physiology, biochemical processes, and yield. The use of arbuscular mycorrhizal fungi (AMF) has exhibited beneficial effects on plants during drought. The objective of this study was to analyse the effect of AMF inoculation on two genotypes of potato plants exposed to water stress, and the photosynthetic traits, enzymatic antioxidant activity, and exudation of low-molecular-weight organic acids (LMWOAs) of potato plants inoculated with two strains of AMF, *Claroideoglomus claroideum* (CC) and *Claroideoglomus lamellosum* (HMC26), were evaluated. Stomatal conductance exhibited a similar trend in the CC and HMC26 treatments for both potato genotypes; moreover, the photosynthetic rate significantly increased by 577.9% between the 100% soil humidity (S0) and 40% soil humidity (S2) stress levels for the VR808 genotype under the CC treatment. The activities of the enzymes catalase (CAT) and ascorbate peroxidase (APX) showed similar trends. In this study, there were different responses among genotypes and treatments. Inoculation with CC under S2 stress levels is a promising potential approach for improving potato growth under drought conditions.

## 1. Introduction

Potato (*Solanum tuberosum*) is one of the most widely cropped plant species globally because it is one of the most important stable foods in the global diet due to its beneficial nutritional impact [1,2]. Worldwide, the production of potato tubers is approximately 380 million tons per year, and a total of 20 million hectares are cultivated with potato [3]. Climate is one of the most important factors in agricultural production; unfortunately, global climate change can cause increases in temperature, changes in precipitation patterns, and increases in evapotranspiration, which can change the water cycle as well as agricultural activity, posing a major challenge to sustainable crop production [4,5,6]. Nonetheless, a variety of abiotic factors pose a serious threat to potato productivity, leading to a reduction in yield. These abiotic factors include drought, temperature, salinity, light, nutrients, flooding, and phytotoxic compounds [7], and drought is the major abiotic constraints on potato productivity, affecting potato physiology and biochemical processes and ultimately reducing yield [8,9]. When water deficiency occurs during the tuber growth period, the yield decreases to a greater extent than when water limitation occurs during other stages of potato growth [10]. Moreover, numerous studies have reported that drought reduces photosynthetic activity via stomatal or nonstomatal limitations or by reducing the intercellular CO_2_ concentration via stomatal restriction and decreasing the chlorophyll content via nonstomatal restriction [10,11,12]. Moreover, there is an increase in antioxidant enzyme activity as a defence mechanism against reactive oxygen species (ROS), which is important since ROS induce membrane lipid peroxidation, damage the membrane system, and even promote the exudation of organic acids through the roots to improve nutrient uptake [13,14].

Arbuscular mycorrhizal fungi (AMF) are organisms that form symbioses with the roots of most plant species [15]. AMF exert several beneficial effects, and one of the most noticeable benefits is increasing the water supply since the hyphae of AMF can act as a complementary root system [16]. In addition, Fritz et al. [17] reported that inoculating potato crops with AMF has a beneficial effect on secondary metabolism, as indicated by increases in phenolic compounds and antioxidant activity. Moreover, Cayún et al. [18] reported similarities between potato plants inoculated with AMF and control plants with respect to the photosynthetic rate and an increase in the concentration of chlorophyll in AMF-inoculated samples. AMF inoculation reportedly increases enzymatic antioxidant activity in corn, olive, and potato leaves and roots [19,20,21]. AMF root colonisation also causes changes in root exudates, increasing the contents of low-molecular-weight organic acids (LMWOAs) secreted by maize and potato plants [18,22]. Nevertheless, little is known about the effect of AMF inoculation on the metabolism of potato plants affected by drought. It has been reported that AMF colonisation can improve drought stress tolerance in apple, chickpea, and tomato plants [23,24,25]. In the case of potato plants, Valdebenito et al. [26] reported that AMF colonisation in potato crops increased water stress tolerance, resulting in increased phenolic content and antioxidant activity in leaves.

According to these findings, we hypothesised that the application of AMF to *Solanum tuberosum* improves defence mechanisms, demonstrated by increased enzyme activity and the exudation of LMWOAs, when plants are grown under drought stress. The main aim of this study was to analyse the effect of AMF inoculation on the physiological and antioxidant responses of potato plants exposed to water stress.

## 2. Results

### 2.1. Photosynthesis Traits

**Figure 1:**
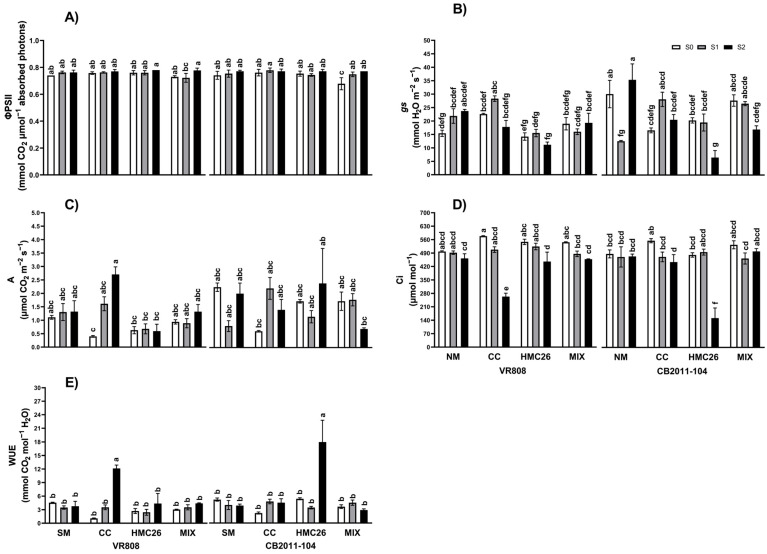
Photosynthetic traits measured in leaves of two genotypes of *Solanum tuberosum* plants inoculated with arbuscular mycorrhizal fungi (AMF) and growing under normal irrigation and two drought conditions. (**A**) Quantum yield of PSII (ΦPSII); (**B**) stomatal conductance (gs); (**C**) photosynthetic rate (A); (**D**) internal CO_2_ concentration (Ci); (**E**) water use efficiency (WUE). Here, NM: non-inoculated plants, CC: plants inoculated with the fungus *Claroideoglomus claroideum*, HMC26: plants inoculated with the fungus *Claroideoglomus lamellosum*, MIX: CC + HMC26; S0: 100%, S1: 70%, S2: 40% of water-holding capacity levels; VR808: yellow skin and yellow flesh genotype, CB2011-104: purple skin and purple flesh genotype. Different letters indicate significant differences according to Tukey’s test (*p* < 0.05).

The quantum yield values (ΦPSII) (Figure 1A) were similar across all treatments, ranging from 0.678 to 0.780 mmol CO_2_ μmol^−1^ absorbed photons. The stomatal conductance (gs) values (Figure 1B) exhibited a similar trend in the *Claroideoglomus claroideum* (CC) and *Claroideoglomus lamellosum* (HMC26) treatments for both potato genotypes. The gs of the CC treatment exhibited an increase at the S1 stress level (70% soil humidity) and a subsequent decrease at the S2 level (40% soil humidity), while in the HMC26 treatment, gs remained the same at the S1 stress level and decreased at the S2 level, with the above decrease being significant only for the treatments of the CB2011-104 genotype compared to the treatment with 100% soil humidity (S0). Specifically, for the CC treatment, in the VR808 genotype, the increase in S1 was 25.5%, and the decrease in S2 was 21.6%. While in CB2011-104, the increase in S1 was 69.9%, and although there was also an increase in S2, it was only 23.6%; for the HMC26 treatment in S2, the decreases were 21.4% for the VR808 genotype and 67.9% for the CB2011-104 genotype, all compared to S0. Moreover, the gs in the control treatments without mycorrhizal fungi (NM) treatment, the VR808 genotype tended to increase when drought was more severe with increases of 41.7% in S1 and 53.6% in S2 compared to S0. Moreover, for the CB2011-104 genotype, the gs decreased significantly (58.2%) at the S1 stress level but increased (17.7%) at the S2 stress level compared to S0. Regarding the photosynthetic rate (A) (Figure 1C), there was a significant increase of 577.9% between the S0 and S2 stress levels for the CC treatment of the VR808 genotype. NM treatment did not significantly affect any of the potato genotypes under drought stress. The internal concentrations of CO_2_ in the leaves (Ci) (Figure 1D) of both genotypes significantly decreased with the individual use of mycorrhizae at the S2 stress level compared to the S0 level, representing a decrease of 54.4% in the VR808 genotype with CC inoculation and 68.3% in the CB2011-104 genotype with HMC26 inoculation. In terms of water use efficiency (WUE) (Figure 1E), compared with the S0 stress level, at the most severe stress level (S2) for genotype VR808, there was a significant increase (1027.9%) with CC inoculation, while for genotype CB2011-104, there was a significant increase (233.5%) with HMC26 inoculation.

### 2.2. Chlorophylls and Carotenoids

**Figure 2:**
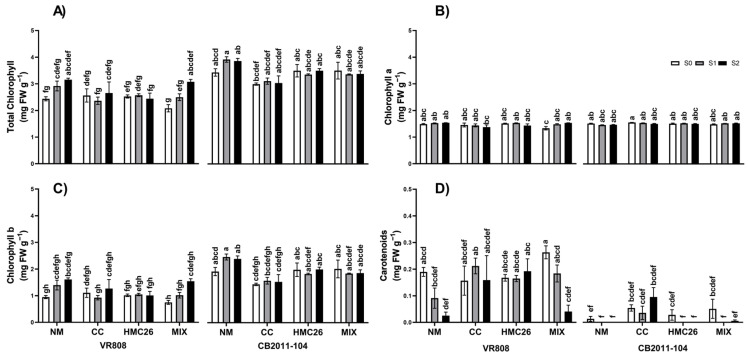
Concentrations of photosynthetic pigments of two genotypes of *Solanum tuberosum* plants inoculated with arbuscular mycorrhizal fungi (AMF) and grown under normal irrigation and two drought conditions. (**A**) Total chlorophyll; (**B**) chlorophyll a; (**C**) chlorophyll b; (**D**) carotenoids. Here, NM: non-inoculated plants, CC: plants inoculated with the fungus *Claroideoglomus claroideum*, HMC26: plants inoculated with the fungus *Claroideoglomus lamellosum*, MIX: CC + HMC26; S0: 100%, S1: 70%, S2: 40% of water-holding capacity levels; VR808: yellow skin and yellow flesh genotype, CB2011-104: purple skin and purple flesh genotype. Different letters indicate significant differences according to Tukey’s test (*p* < 0.05).

Regarding total chlorophyll (Figure 2A), in general, the CB2011-104 genotype presented higher concentrations than the VR808 genotype, with concentrations in the ranges of 3.0–3.9 mg g^−1^ and 2.1–3.2 mg g^−1^, respectively. HMC26 inoculation resulted in a stable total chlorophyll concentration in both genotypes, while for the remaining treatments, there were no clear trends. Chlorophyll a (Figure 2B) did not differ between treatments or genotypes. In contrast, chlorophyll b (Figure 2C) showed the same trend as the total chlorophyll. With respect to the carotenoid concentrations (Figure 2D), for the VR808 genotype, the concentrations tended to decrease by 86.8% and 84.4% in the NM and MIX (CC + HMC26) inoculation treatments, respectively, under the S2 drought stress level. For the CB2011-104 genotype, due to the low concentrations obtained, there were no clear trends attributable to any experimental factors.

### 2.3. Enzymatic Antioxidant Activity

**Figure 3:**
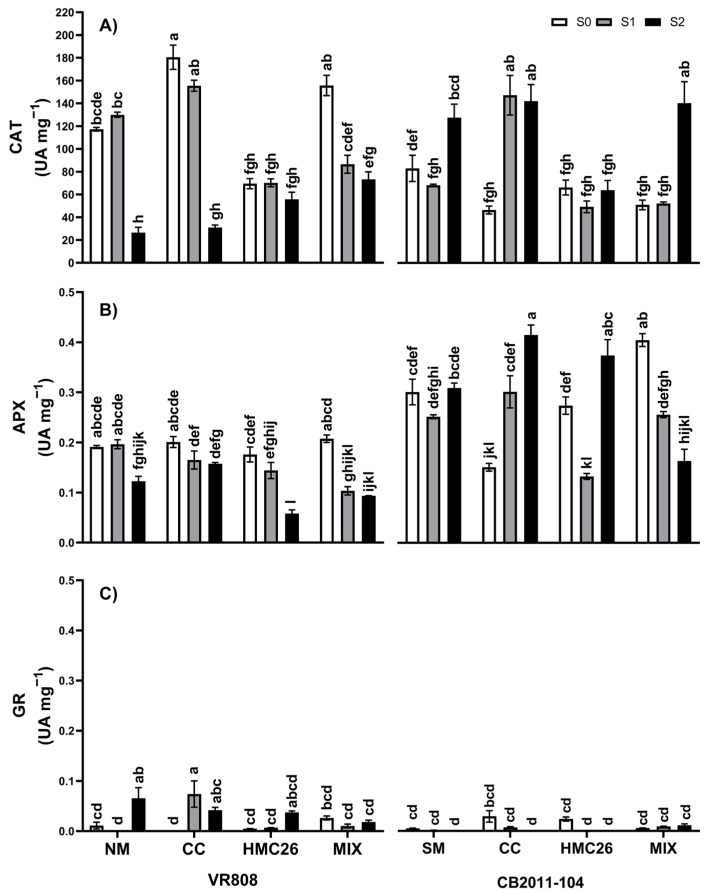
Antioxidant enzymatic activity levels of two genotypes of *Solanum tuberosum* plants inoculated with arbuscular mycorrhizal fungi (AMF) and grown under normal irrigation and two drought conditions: (**A**) Catalase (CAT) enzyme; (**B**) ascorbate peroxidase (APX) enzyme; (**C**) glutathione reductase (GR) enzyme. Here, NM: non-inoculated plants, CC: plants inoculated with the fungus *Claroideoglomus claroideum*, HMC26: plants inoculated with the fungus *Claroideoglomus lamellosum*, MIX: CC + HMC26; S0: 100%, S1: 70%, S2: 40% of water-holding capacity levels; VR808: yellow skin and yellow flesh genotype, CB2011-104: purple skin and purple flesh genotype. Different letters indicate significant differences according to Tukey’s test (*p* < 0.05).

Regarding catalase (CAT) enzyme activity (Figure 3A), in the VR808 genotype, there were significant decreases in CAT activity in the NM, CC, and MIX inoculation treatments, especially at the S2 drought stress level, with respect to that at the S0 level, with decreases of 77.4%, 82.8%, and 52.9%, respectively. In the CB2011-104 genotype, the opposite trend was observed, where the CAT activity levels of these treatments increased at the S2 stress level, with increases of 53.6%, 205.4%, and 175.2%, respectively, compared to that of the control. With respect to the ascorbate peroxidase (APX) enzyme activity (Figure 3B), there was a similar trend to that of CAT activity, where the APX activity tended to decrease under the most severe stress conditions with respect to that under the S0 level in the VR808 genotype, with significant decreases of 35.6%, 67.1%, and 54.8% in the NM, HMC26, and MIX inoculation treatments, respectively. However, in the CB2011-104 genotype, there were significant increases in APX activity at the S2 level only in the CC and HMC26 inoculation treatments, with values of 173.6% and 36.6%, respectively. Finally, the glutathione reductase (GR) enzyme activity (Figure 3C) did not show clear trends due to the low activity values obtained.

### 2.4. Concentrations of Low-Molecular-Weight Organic Acids

**Figure 4:**
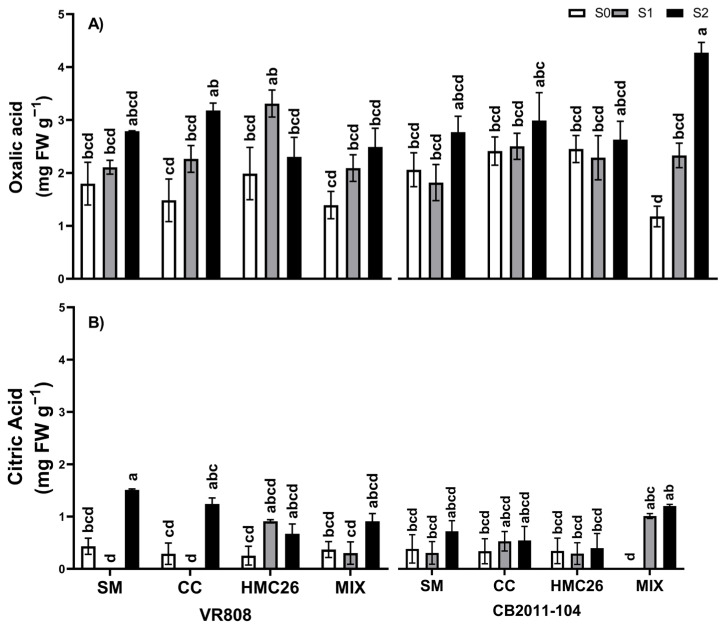
Concentrations of low-molecular-weight organic acids (LMWOAs) in the rhizosphere of two genotypes of *Solanum tuberosum* plants inoculated with arbuscular mycorrhizal fungi (AMF) and grown under normal irrigation and two drought conditions: (**A**) Oxalic acid; (**B**) citric acid. Here, NM: non-inoculated plants, CC: plants inoculated with the fungus *Claroideoglomus claroideum*, HMC26: plants inoculated with the fungus *Claroideoglomus lamellosum*, MIX: CC + HMC26; S0: 100%, S1: 70%, S2: 40% of water-holding capacity levels; VR808: yellow skin and yellow flesh genotype, CB2011-104: purple skin and purple flesh genotype. Different letters indicate significant differences according to Tukey’s test (*p* < 0.05).

Two LMWOAs were identified and quantified (Figure 4). Specifically, oxalic acid (Figure 4A) exhibited a greater concentration than citric acid and tended to increase under the most severe drought stress (S2), showing significant differences between the VR808 genotype inoculated with CC and the CB2011-104 genotype with MIX inoculation, exhibiting increases of 114.5% and 262.8%, respectively, when comparing the same mycorrhizal treatment at the S0 level. Moreover, the unique treatment that did not follow this behaviour was the VR808 genotype inoculated with HMC26, where there was an increase at the S1 drought stress level but a decrease at the S2 drought stress level. Additionally, citric acid (Figure 4B), in general, presented the same trend as oxalic acid.

### 2.5. Multivariate Analysis

**Figure 5:**
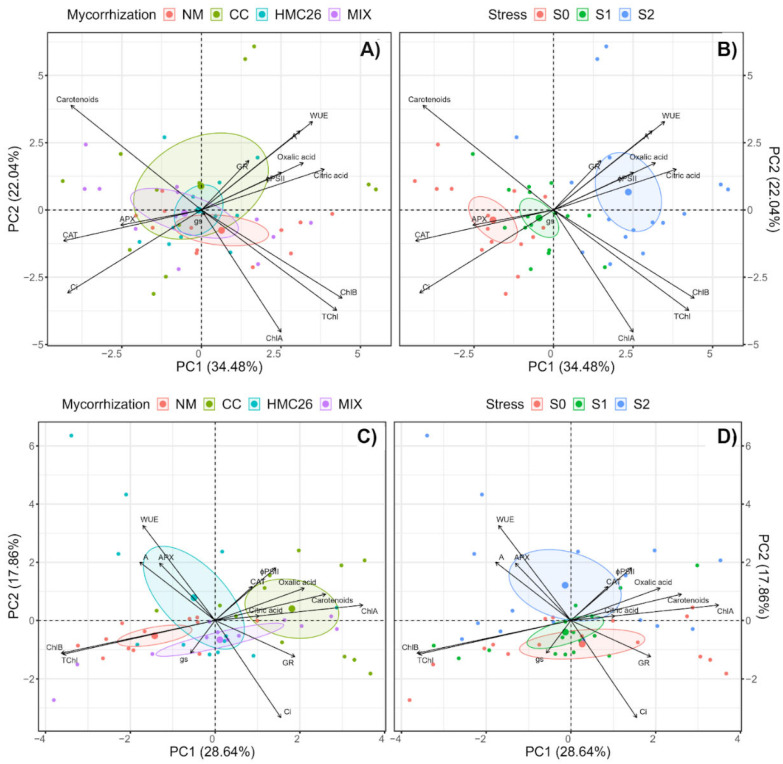
Principal components (PCs) for the experimental variables determined in two genotypes of *Solanum tuberosum* plants inoculated with arbuscular mycorrhizal fungi (AMF) and grown under normal irrigation and two drought conditions. The graph shows the experimental individuals according to PC grouped according to the (**A**) mycorrhizal treatments of genotype VR808; (**B**) water stress level of genotype VR808; (**C**) mycorrhizal treatments of genotype CB2011-104; and (**D**) water stress level of genotype CB2011-104. Here, NM: non-inoculated plants, CC: plants inoculated with the fungus *Claroideoglomus claroideum*, HMC26: plants inoculated with the fungus *Claroideoglomus lamellosum*, MIX: CC + HMC26; S0: 100%, S1: 70%, S2: 40% of water-holding capacity levels; VR808: yellow skin and yellow flesh genotype, CB2011-104: purple skin and purple flesh genotype. ΦPSII: Quantum yield of photosystem II, gs: Stomatic conductance, A: Photosynthetic rate, Ci: Leaf internal CO_2_ concentration, WUE: Water use efficiency, TChl: Total chlorophyll, ChlA: Chlorophyll a, ChlB: Chlorophyll b, GR: Glutathione reductase enzyme activity, CAT: Catalase enzyme activity, APX: Ascorbate peroxidase enzyme activity.

A factor analysis by means of principal component analysis performed for the VR808 potato genotype allowed us to associate inoculation with AMF with specific experimental variables depending on the type of inoculum (Figure 5A). In detail, the CC strain was associated with high levels of photosynthetic traits as ΦPSII, WUE, GR enzymatic activity, and low-molecular-weight organic acid exudation. The NM, HMC26, and MIX treatments were mainly associated with gs, and an association with high levels of the other variables studied here was unclear, remarking the importance of the CC strain in the stress response of the VR808 genotype. Regarding the effect of drought stress (Figure 5B), the S2 level was mainly associated with the same parameters as those previously described for CC inoculation. For the CB2011-104, which is a purple-coloured potato genotype, regarding the effect of AMF inoculation (Figure 5C), the CC strain was also strongly associated with enzymatic and photosynthetic traits and pigments; variables such as CAT activity, ΦPSII, chlorophyll a, and carotenoids; and the exudation of both LMWOAs, whereas the HMC26 strains are related to the APX activity and photosynthetic traits such as A and WUE. Regarding the drought stress factor (Figure 5D), the S2 level was also strongly associated with the variables mentioned above, including strong associations with the APX and CAT activities.

## 3. Discussion

Improved photosynthetic behaviour is directly associated with increased crop yields [27]; however, abiotic stresses such as drought limit the efficiency of the photosynthetic apparatus by damaging the thylakoid membrane, reducing the contents of photosynthetic pigments, and negatively affecting the function of PSII by reducing the quantum yield [28]. In a previous study in which the effects of fungicides without water deficit were evaluated on the same genotypes analysed here, there were no differences in ΦPSII between genotypes [18]; furthermore, water deficit in other potato genotypes has not shown a noticeable influence on this trait [29]. The symbiotic association generated by AMF regulates various processes in plants, such as stomatal aperture through abscisic acid metabolism, improving gas exchange, transpiration rate, stomatal conductance, and improving root efficiency under water stress conditions [30]. Moreover, gs can be associated with the water status of the plant since it measures stomatal opening; therefore, if gs decreases, there is less water loss due to stomatal closure [31]. In other potato varieties, gs also decreases when water stress is more severe [29]. Regarding the role of AMF symbiosis, in *Terminalia arjuna* plants inoculated with AMF, a decrease in gs was reported when water stress was applied [32], although the opposite effect has also been shown in *Vitis vinifera* leaves [33]. In *Solanum lycopersicum* plants subjected to mild and moderate drought stress, gs decreases significantly with respect to that of the control [34]. The photosynthetic rate of plants subjected to AMF inoculation has been shown to increase in *Solanum nigrum* and *Digitaria sanguinalis* plants [35], as well as in *Terminalia arjuna* plants subjected to water stress [31]. In contrast, in previous studies using wheat plants subjected to water stress in which the CC fungus was used, no significant differences were detected in the photosynthetic rate with the application of stress [36]. Increases in WUE have been reported in *Cinnamomum migao* plants [37], *Terminalia arjuna* plants [32], and some wheat varieties [36] with the application of AMF and water starvation. On the other hand, in potato plants infected with potato virus Y and inoculated with the AMF *Funneliformis mosseae* and *R. irregularis*, improvements in photosynthetic parameters were observed in both healthy and infected plants, although *F. mosseae* was shown to improve these parameters slightly more [38].

Chlorophylls play major roles in photosynthesis because they function as light-harvesting antenna pigments and electron transfer cofactors [39]. On the other hand, carotenoids absorb light energy and transfer it to chlorophylls, in addition to absorbing and releasing excess light energy [40]. Responses to water limitation have varied depending on the type of plant studied, with increases and decreases observed in the concentrations of these photosynthetic pigments [41,42,43]. Specifically, in comparing the results reported here, considering or not considering AMF inoculation in addition to water starvation, with the results previously reported by Cayún et al. [18], who studied the same potato genotypes but without the application of drought, higher concentrations of total chlorophyll were found for all treatments. Moreover, regarding water stress and inoculation with AMF, in *trifoliate orange* plants, AMF significantly increased the contents of photosynthetic pigments when water stress was applied to the soil compared to non-inoculated plants [44]. Similar results were observed in *Vitis vinifera* L. plants, which showed a significant increase in the contents of photosynthetic pigments due to the combination of AMF inoculation and water stress [33]. Regarding the carotenoid concentrations, for the VR808 genotype, the concentration levels tended to decrease by 86.8% and 84.4% in the NM and MIX treatments, respectively, under the S2 drought level compared to those in the same inoculation treatment without stress (S0). In contrast, meta-analyses have shown that concentrations increase with inoculation and the application of water stress and that the percentage of this increase depends on the level of stress applied [45].

Regarding enzymatic antioxidant activities, previous studies have evaluated the effect of AMF inoculation on the response to water stress, showing that in *Bombax ceiba*, inoculation with AMF under water shortage increased CAT, APX, and GR activities by 318.5%, 34.1%, and 22.8%, respectively [46], whereas in walnut, these activities were increased by 340.4%, 106.3%, and 77.2%, respectively, compared to those in non-inoculated plants, and these increases were significant for CAT and APX [47]. Moreover, in tomato plants subjected to water and heat stresses, CAT activity increased by 42% and 57%, respectively, when plants were inoculated with two strains of AMF [48]. In maize plants, APX and CAT activities also increase upon water stress and as the stress becomes more severe [49]. In *Satureja hortensis* plants, there is also an increase in enzymatic antioxidant activity due to drought stress [42]. Although most studies have shown an increase in enzyme antioxidant activity under water stress upon inoculation with mycorrhizae, we observed that the effect varied according to the potato genotype. Valdebenito et al. [26], using similar experimental designs to those used in this study, evaluated lipid peroxidation through TBARS to analyse the damage associated with water stress and observed that for the VR808 genotype in the NM, CC, and MIX inoculation treatments, there was a tendency for lipid peroxidation to increase with both levels of stress, which was significant in the CC-S2 treatment, while in the HMC26 treatment, there was a decrease with stress, which was significant in the S2 treatment. However, for the CB2011-104 genotype in the NM and CC inoculation treatments, there were no significant differences with stress; in the HMC26 inoculation treatment, there was a significant increase at the S1 level, and in the MIX treatment, there was a significant decrease at the S1 and S2 levels. In potato leaves, where they measured CAT, peroxidase (POX), and APX activities, there was an increase in the activities when inoculated with the mycorrhizal *Glomus intraradices* compared to the non-inoculated treatment. However, the increase varied depending on the potato variety (Arinda, Agria and Santé) [21]. In potato tubers where two AMF strains (*C. claroideum* and *C. lamellosum*) were used, there were improvements in non-enzymatic antioxidant activity, being higher with the *C. lamellosum* strain [50].

The root exudation of LMWOAs is a well-recognised mechanism for modifying the plant rhizosphere due to its ability to solubilise and mobilise nutrients such as P, sequester phytotoxic elements, and provide a labile carbon source to soil microorganisms [51,52]. Previously, oxalic acid was reported to be present at concentrations between 0.55 and 1.53 mg g^−1^ in the potato genotypes also used this study [18], and it was lower than the concentrations reported in the present study. The presence of AMF in *Solanum lycopersicum* plants has been shown to increase the contents of LMWOAs such as oxalic, succinic, and citric acids [53], and in corn plants, an increase in the exudation of these compounds due to inoculation has been reported [54]. For instance, in a study evaluating the effects of the AMF *Rhizophagus aggreatus*, *Claroideoglomus etunicatum*, and *Funneliformis mosseae* on corn plants, 10 LMWOAs were isolated and identified, but the compositions of the exudates varied depending on the growth stage and the fungal strain used as inoculant [55]. On the other hand, there is no recent information about the effect of the use of AMF under water stress on the composition and levels of organic acids exuded to the rhizosphere, but its overproduction under other stresses, such as Cd stress [56] and salt stress, as well as its accumulation in plants inoculated with AMF [57], suggests its main role in the improvement of environmental conditions, allowing efficient plant establishment and growth.

In conducting a global analysis of experimental variables and depending on the potato genotype used, it was possible to distinguish different responses, especially in the case of the enzymatic antioxidant response in relation to water starvation. In another study with Hopehely and Demon, both of which are uncoloured potato genotypes, different responses of the enzymatic antioxidants were observed; the highest enzyme activities were reported in Hopehely plants growing under stress, while in Demon plants, the highest activities were found in the unstressed treatments [58]. The above findings suggest that different potato genotypes respond differently to water stress, which is also a useful indicator for classifying tolerant and sensitive genotypes [59]. Moreover, for the two potato genotypes used here, the CC strain had a great influence on the CB2011-104 genotype, but the HMC26 strain also had an influence, although to a lesser extent and with different experimental variables than the CC strain. Previously, Cayún et al. [18], working with the same genotypes used here under AMF inoculation but without the influence of water stress, reported no clear correlations that allowed them to establish the effect of the strains used on the characteristics measured. In other studies, it has been shown that there is no single AMF strain that positively influences all the different varieties but consistently improves some desirable characteristics, suggesting that the best AMF–plant host combination should be analysed and identified [60,61]. Previously, Valdebenito et al. [26], who worked with the same genotypes and AMF treatments under similar water stress conditions, determined phenolic compounds and non-enzymatic antioxidant activity and found that for the VR808 genotype, a better interaction at the S1 stress level was exhibited under inoculation with CC and HMC26 individually, while for the CB2011-104 genotype, the best interaction was at the S2 stress level, independent of the mycorrhizal treatments. On the other hand, both studied genotypes exhibit distinct characteristics, with the VR808 genotype having yellow flesh and skin and the CB2011-104 genotype having purple flesh and skin, resulting in different secondary metabolites in both leaves and tubers, which leads to varied metabolic responses [17,60]. Therefore, despite being positively influenced by CC, the impacted parameters differ.

AMF regulate different biochemical mechanisms in plants under water stress, including the elimination of ROS by increasing water content through increased uptake through hyphae [62]. Also, AMF can increase enzymatic and non-enzymatic antioxidants and enhance the levels of heat shock transcription factors (Hsfs), which play an important role in regulating signal transduction and genetic response to stress to maintain the balance of ROS produced under the stress condition [62,63]. For example, in basil crops under salt stress conditions, an increase in antioxidant enzyme activity was observed, followed by a decrease in their activity, showing that the effect of AMF on antioxidant enzymes varies depending on the specie and also the AMF strain [64,65].

Our results indicate that the responses of the evaluated characteristics varied depending on the genotype, where for photosynthetic traits, the individual use of AMF (CC) and S2 stress levels showed the most notable improvements, while for the enzymatic antioxidant activity, there were different responses among the genotypes, where CB2011-104 showed a tendency to increase these activities as the stress increased, while in VR808, the opposite behaviour was observed. Finally, the exudation of LMWOAs was affected by both AMF and stress levels and increased with the use of CC as a mycorrhizal treatment and by the S2 stress level. There are no previous studies on the effect of water stress on the tubers of potato plants grown under water stress, but Valdebenito et al. [26] previously reported that in the leaves of these genotypes, under the same mycorrhizal and stress treatments, the use of CC and HMC26 mycorrhizae can protect potato plants against water stress and that this protection varies according to the genotype and mycorrhizal inoculant used, observations that are strongly supported by the results presented here.

## 4. Materials and Methods

### 4.1. Reagents

Water (HPLC grade), acetone, hydrogen peroxide (30%), phosphoric acid (85%), monopotassium phosphate (KH_2_PO_4_), dipotassium phosphate (K_2_HPO_4_), calcium chloride (CaCl_2_), ethylenediaminetetraacetic acid (EDTA), albumin fraction V from bovine serum (BSA), and glutathione (GSSG) were obtained from Merck (Darmstadt, Germany). Urea and oxalic acid and citric acid standards were obtained from Supelco (Bellefonte, PA, USA). Bradford reagent and β-nicotinamide adenine dinucleotide phosphate (NADPH) (≥93%) were obtained from Sigma–Aldrich (Steinheim, Germany). Polyvinylpyrrolidone (PVP) was obtained from Winkler (Lampa, Santiago, Chile).

### 4.2. Potato Samples and Biological Materials

A completely randomised factorial design of 3 × 2 × 4 was used, incorporating the following as the first experimental factors: (1) plants without inoculation (NM), (2) plants inoculated with *Claroideoglomus claroideum* (CC), (3) plants inoculated with *Claroideoglomus lamellosum* (HMC26), and (4) plants inoculated with MIX: CC + HMC26. For each level of inoculation, we used three watering regimes: (i) well-watered conditions (90–100% of available water capacity, S0), (ii) plants subjected to mild water stress (70% of available water capacity, S1), and (iii) plants subjected to severe water stress (40% of available water capacity, S2). Additionally, two potato genotypes (*Solanum tuberosum*) were grown, VR808: white potato and CB2011-104: purple potato. In total, there were 24 treatments with 3 replications each (n= 72).

The plants were grown in 11 L pots in the greenhouse of the Departamento de Ciencias Químicas y Recursos Naturales, Universidad de La Frontera, Temuco (38°44′49.671″ S, 78°36′53.337″ W). Seeds of both potato genotypes with different skin and pulp colours were supplied by Papas Arcoiris Ltda (Puerto Varas, Chile). The plants were maintained under 50% artificial shade using plastic mesh in the greenhouse with a light/dark photoperiod of 16/8 h and a day/night temperature of 25/18 °C. At sowing, inoculation was carried out with each mycorrhizal inoculum, which was added to the substrate below the potato tubers at a rate of 5 g/pot (approximately 700 spores/g) [17]. Non-inoculated plants received the same amount of autoclaved mycorrhizal inoculum along with a 10 mL aliquot of a filtrate (on Whatman N°1 paper) of both AM inocula to provide a general microbial population free of AM fungal propagules. Plants inoculated with one inoculum also received the filtrate of the other inoculum.

Additionally, the plants were fertilised with nitrogen, phosphorus, and potassium doses recommended by the INIA potato cultivation manual [66], using 0.45 g N/plant as urea, 0.28 g P_2_O_5_/plant, and 0.35 g K_2_O/plant as sources, and the commercial fungicide REFLECTXTRA^®^ was added to all treatments for agronomic management. After sowing, all plants were subjected to regular irrigation until the beginning of tuber formation, when water stress was applied under the three conditions mentioned earlier. The substrates used were soil and sand in a 2:1 ratio and were autoclaved at 121 °C and 1 atm pressure for 20 min. Potato leaves were harvested three times, at 60 days after sowing (DAS), 90 days, and finally at 120 days, and then stored at −80 °C until analysis.

### 4.3. Photosynthetic Parameter Determination

The photosynthetic parameters were measured using Targas-1 equipment (PP Systems, Amesbury, MA, USA). The determined variables corresponded to the internal concentration of CO_2_ (Ci: μmol mol^−1^), photosynthesis rate (A: μmol CO_2_ m^−2^ s^−1^), stomatal conductance (gs: mmol H_2_O m^−2^ s^−1^), and water use efficiency (WUE: mmol CO_2_ mol^−1^ H_2_O). Moreover, the efficiency of PSII (ΦPSII) was evaluated using portable Fluorpen equipment (Photon Systems Instruments, Drasov, Czech Republic) with Fluorpen 1.0 software. Photosynthetic variables were measured 2 h after the onset of the photoperiod using the second youngest leaf.

### 4.4. Chlorophyll and Carotenoid Contents in Leaves

The contents of total chlorophyll, chlorophyll a, chlorophyll b, and carotenoids were estimated. Leaf samples were crushed with liquid nitrogen; 100 mg of the sample was weighed, dissolved in 80% acetone, stirred, and finally incubated at 4 °C for 24 h. Afterward, the samples were centrifuged at 1000× *g* at 4 °C for 15 min, and the absorbance was read at 664, 647, and 430 nm in a Synergy HTX UV–visible microplate spectrophotometer (BioTek, Winooski, VT, USA) [67,68]. The pigment concentrations were determined according to the mathematical models presented by Lichtenthaler [67].

### 4.5. Evaluation of Enzyme Antioxidant Activities

The quantification of total proteins was performed using the Bradford method [69] adapted to microplates, and the measurement of the enzymatic antioxidant activities of catalase (CAT), ascorbate peroxidase (APX)s and glutathione reductase (GR) was carried out according to the methods described by Cayún et al. [18]. For the enzymatic extracts, 0.3 g of vegetal sample was crushed using liquid nitrogen, dissolved in 0.9 mL of 0.1 M potassium phosphate buffer (pH 7.5), centrifuged (Centurion Scientific Ltd, Bosham, United Kingdom) at 13,000× *g* and 4 °C for 15 min, and stored at −20 °C until analysis. Then, 10 mg of PVP was added to 0.4 mL of extract for CAT, APX, and GR, and it was centrifuged at 12,000× *g* and 4 °C for 10 min. The supernatant was used for enzymatic determinations, and the results were expressed as specific activity (mol AU/mg protein). For all determinations, a Synergy HTX UV–visible microplate spectrophotometer (BioTek, Winooski, VT, USA) was used.

### 4.6. Concentrations of Low-Molecular-Weight Organic Acids

The extraction of LMWOAs was carried out based on the method described by Cayún et al. [18] with minor modifications; 0.5 g of rhizosphere soil was crushed and dissolved in 1 mL of 0.2 M calcium chloride, shaken, and centrifuged (Centurion Scientific Ltd, Bosham, UK) at 4000× *g* at 4 °C for 15 min. Afterward, the supernatant was filtered through 0.45 μm filters. The extraction procedure was repeated three times.

Chromatographic separation was performed using high-performance liquid chromatography with diode array detection (HPLC-DAD) (Shimadzu, Tokyo, Japan) equipped with a quaternary pump (LC-20AD), a degassing unit (DGU-20A5R), a column oven (CTO-20A), an autosampler (SIL-20A), and a UV–Vis diode array detector (SPD-M20A) using a C_18_ column (Eclipse, 250 × 4.6 mm, 5 µm) and a C_18_ precolumn (NovaPak, Waters, 22 × 3.9 mm, 4 µm). The mobile phase was 0.2 N phosphoric acid (pH 2.1) at a flow rate of 1.0 mL min^−1^ with isocratic elution at 30 °C. Detection was performed at 210 nm with an analysis time of 15 min using oxalic acid (y = 12,295x + 11,179, detection limit (DL): 1.90 mg L^−1^, quantification limit (QL): 6.34 mg L^−1^, linear range (LR): 6.34 to 100 mg L^−1^) and citric acid (y = 1274.2x + 3763.9, DL: 0.20 mg L^−1^, QL: 0.66 mg L^−1^, LR: 0.66 to 500 mg L^−1^) as external calibration standards.

### 4.7. Statistical Analysis

All the statistical analyses and figure generation were performed using R version 4.3.0. Three-way ANOVA was used to test for significant differences between the measurements of each experimental variable, with the factors ‘Genotype’, ‘Mycorrhization’, and ‘Stress’ as sources of variation. For the variables with significant differences, the means were compared using Tukey’s HSD multiple range test with the ‘agricolae’ package version 1.3.5. Moreover, the dataset was split at the ‘Genotype’ level and subjected to principal component analysis (PCA). Confidence ellipses (group means) for ‘Mycorrhization’ and ‘Stress’ were generated using the ‘FactoMineR’ package version 2.7 and ‘factoextra’ version 1.0.7.

## 5. Conclusions

We evaluated whether the application of AMF to *Solanum tuberosum* plants improved the photosynthetic level defence, enzymatic antioxidant activity, and LMWOA exudation against water starvation. We observed that both potato genotypes inoculated with *Claroideoglomus claroideum* individually and under the most severe drought level (S2) exhibited the greatest influence based on the determinations analysed; however, depending on the genotype tested (VR808 and CB2011-104), the parameters varied according to these factors, which may be due to the different characteristics presented by both plant materials. Therefore, further studies are needed to understand the beneficial effects of the individual use of AMF against water stress on different *Solanum tuberosum* genotypes.

## Data Availability

The data presented in this study are available upon request from the corresponding author.
